# Polypharmacy - is there a cure for drug therapy problems?

**DOI:** 10.3325/cmj.2023.64.295

**Published:** 2023-08

**Authors:** Helena Orehovački, Andrea Brajković, Lucija Ana Bićanić, Iva Mucalo

**Affiliations:** 1 Health Care Center Zagreb-Centar, Zagreb, Croatia; 2 Faculty of Pharmacy and Biochemistry, University of Zagreb, Zagreb, Croatia *abrajkovic@pharma.hr*

## Simple challenge – complex problem

The use of pharmaceuticals is the most common medical intervention, possessing immense potential for achieving both positive and negative outcomes (1). While pharmaceuticals dominantly provide benefits, sometimes the risks associated with a particular drug outweigh its advantages (2). Drug therapy problems (DTPs) encompass adverse events encountered by a patient during drug treatment that either currently or potentially hinder the attainment of desired therapeutic goals, necessitating the expertise of health care professionals (3). Examples of DTPs include the employment of unneeded or ineffective medications, the lack of prescribed medications, adverse drug reactions (ADRs), and medication non-adherence (4). The significance of DTPs in health care cannot be underestimated due to their grave consequences, such as diminished quality of life, escalated health care expenditures, and fatalities associated with medication use (5). In the United States, the projected yearly expenditure attributed to drug-related morbidity and mortality resulting from suboptimal drug therapy surpasses $520 billion (6). These medication errors incur substantial costs and profoundly influence both patient well-being and the overall health care system (1). As the population ages, an increasing number of individuals consume multiple medications and are thus at greater risk of experiencing DTPs (4,7). This phenomenon particularly affects the elderly population, which is most vulnerable to the escalation of medication usage and the associated repercussions (8).

The World Health Organization defines polypharmacy as the concurrent administration of numerous drugs or the administration of an excessive number of drugs (9). To establish a threshold for polypharmacy, a cut-off value of five or more medicines was proposed, as this number is associated with the highest incidence of adverse outcomes (10,11). Although polypharmacy in older individuals may be necessary and appropriate, it can also have detrimental effects, including decreased adherence, undesirable drug reactions, increased health care utilization, falls, cognitive dysfunction, and death rate, ultimately leading to unfavorable health outcomes (7,11,12).

## Polypharmacy and medication mismanagement

Current medical practice guidelines often stipulate the use of multiple medications to manage chronic diseases and achieve optimal clinical benefits. Consequently, approximately one-third of patients are prescribed at least five prescription medications. In the hospital setting, over 40% of patients receive between 5 and 8 medications, while more than 35% receive 9 or more (13-15). In the Croatian ambulatory health care setting where comprehensive medication management (CMM) services were established for older patients with cardiovascular diseases, the average number of medications used per patient was 10.8 ± 3.6 (16). There have been reports of a significant increase in the number of medications prescribed after hospital discharge (14). Both in ambulatory and hospital care settings, around 50% of older patients are prescribed one or more unnecessary drugs and are thus at an increased risk of adverse drug reactions (13,14). Elderly patients, who often present with multiple chronic conditions, are particularly vulnerable to adverse drug reactions due to their frailty, heightened sensitivity to pharmacotherapy, and frequent admissions for acute illnesses (15).

Pharmacists play a crucial role in patient care by using their advanced knowledge and skills to prevent medication-related harm, optimize complex therapeutic regimens, and enhance the overall quality of medication usage processes (1,17). Given their extensive formal education and expertise in both drug therapy and the use of nutritional supplements, pharmacists are uniquely positioned to address adverse effects arising from polypharmacy and inappropriate drug use (2,18).

## Comprehensive medication management services

A way to assure effective and safe medication use is to establish primary health care services focused on drug therapy management. Although fairly novel and insufficiently incorporated in health care systems across Europe, CMM services provided by trained pharmacists can address the problem of medication mismanagement stemming from polypharmacy and inappropriate drug use. The same standardized service model was employed in the USA (19-22) and elsewhere (23-25) and has demonstrated the capacity to improve clinical outcomes (19,22-28) and reduce costs (19,29,30).

CMM is the standard of care that assures that each patient’s medications, both prescribed and nonprescribed, are adequate and effective for the patient, safe considering other medical conditions and drug therapy, and that the patient can and is willing to use them as intended (31). The concept and definition of the CMM services have developed throughout the years. In 2003, Medication Therapy Management (MTM) services, a service disparate from CMM, were suggested by the Centre for Medicare and Medicaid Services. Namely, MTM is a drug therapy optimization approach commonly delivered to patients who fulfill specific criteria defined through Medicare’s rules and regulations, albeit targeting only specific medications. Moreover, the pharmacist providing MTM services works independently from the health care team, while the service is typically offered and operated by a payer (32). The practice of the CMM services, a well-defined method for the medication management provision, was proposed in 2006 by the coalition Patient-Centered Primary Care Collaborative. Unlike MTM, CMM is a patient-centered, sustained, and integrative practice that considers all patients’ health care necessities and concerns. Furthermore, this service is usually carried out by a clinical pharmacist who collaborates closely with the patient and other health care providers with the purpose of optimizing the patient's medication therapy, accompanied by a required follow-up as specified in the care plan (3,33).

## The recognition of the CMM services

As a clinical practice with a foundation in pharmaceutical care, CMM services are the only patient-focused pharmaceutical practice endorsed by several engaged stakeholders within the health care sector, including the Patient-Centered Primary Care Collaborative (31), American College of Clinical Pharmacy (ACCP) (34,35), and Get the Medications Right Institute (36). The ACCP, a professional association for clinical pharmacy in the United States, defined the discipline of clinical pharmacy as a medical service that embraces the philosophy and framework of pharmaceutical care practice in the form of CMM services. It set forth the practice's guidelines and standards for clinical pharmacists in addition to unambiguously outlining the patient's care process and data documentation (37). Furthermore, in 2014 ACCP defined CMM and since then the uniformed implementation of CMM in patient-centered, multidisciplinary care has been endorsed (38).

## The quadruple aim in health care

The concept of triple aim, first described in 2008, includes enhancing population health, improving patients' care experience, and decreasing costs (39). As the increase in burnout among medical specialists and other care providers was acknowledged, these goals were broadened to the quadruple aim to incorporate the additional objective of enhancing the health care providers' professional life (40). Even more, in 2022 the American Medical Association proposed including the fifth aim of promoting health equity (41). Since the introduction of the pharmaceutical care model in the health care system in the 1990s, CMM services have favorably affected clinical, humanitarian, and economic outcomes, hence demonstrating significant effect across all four areas of the quadruple aim (22). CMM services have optimized the health care system’s performance by improving patients' health outcomes, including their clinical outcomes and health-related life quality, enhancing their experience of care, reducing health care costs, and improving health care providers' work-life balance (22). Improving patients' well-being and overall health adds significant input to patients' experience of pharmaceutical care (42). Furthermore, it highlights the exceptional value of the pharmacist providing CMM services as a multidisciplinary health care team member.

The greatest welfare this pharmacist-led service can bring to patients is better chronic disease management and improved health-related quality of life (16,42-44). By ensuring appropriate and effective medications for each patient, decreasing the inadequate and inappropriate medication use, and increasing patients' adherence, CMM allowed better management of chronic diseases such as hypertension, hyperlipidemia, diabetes, cardiovascular diseases, and chronic obstructive pulmonary disease (16,45-48). Moreover, as a result of CMM services, patients experienced enhanced overall well-being and health-related quality of life (42,44,49). Keeping these patients outside of the hospital and reducing emergency department visits tremendously affects health care costs and is important for diverse stakeholders in the health care system (44,45,50). The financial impact of CMM services has been shown by the return-on-investment (ROI) analysis, which quantifies the added CMM's value in comparison with the delivery cost. A positive ROI with an average of 3:1-5:1, up to 12:1, indicates that pharmacist-led clinical services can save $3-12 to the health care system for every $1 invested in their implementation and provision (45).

Furthermore, when CMM services are provided in outpatient care settings, namely ambulatory care, general practitioners find the pharmaceutical care practitioner a valuable partner in the management of patients with complex therapeutic regimens. As they recognize the pharmacist as an expert in medication optimization, they can dedicate additional time to diagnostic and medical care delivery (51-53).

## Croatian experience

Based on international experience and fruitful collaboration with pioneers and experts in the area of pharmaceutical care, CMM services in Croatia were introduced at the primary care level in 2018, in the ambulatory clinic Health Care Center Zagreb – Centar (HCZC). The HCZC’s CMM program was developed in collaboration with the University of Zagreb Faculty of Pharmacy and Biochemistry. This initiative was established to enhance the management of chronic health conditions and maximize the therapeutic benefit of medications. The HZCZ is Croatia's largest primary county health care center, and is the only Croatian health care setting with four CMM practitioners addressing irrational drug use and medication mismanagement, two of whom are fully employed. Namely, following five years of piloting the implementation of CMM services and demonstrating its multiple health benefits (16,49,54,55), the services were in 2022 fully implemented in HCZC. The wider integration of CMM into the Croatian health care system is expected upon the adoption of the revision of the Pharmacy Act. These amendments will set up a legal structure for the CMM and facilitate their reimbursements through the Croatian Health Insurance Fund (CHIF).

## Conclusion

As a foundation of the professional practice of clinical pharmacists, CMM is developed to improve patients' outcomes and their medication experience through a close collaboration of the pharmacist and the patient and other health care team members in an interdisciplinary environment (56). Therefore, if we are to succeed as a profession, clinical pharmacy needs to broaden its use of collaborative drug therapy management agreements and varied collaborative licensing procedures for the CMM delivery (56). When clinical pharmacists have a unique and well-defined professional practice with conceptual and ethical framework, the CMM services will become reality. This scenario will then enable all the clinical pharmacists providing pharmaceutical care to assess and compare their results, and progress from there.

Furthermore, for the CMM services to reach their maximum potential, it is crucial to have an ample number of highly skilled and experienced pharmacist-practitioners, that is, clinical experts eager to enhance their clinical expertise by gaining new knowledge. Moreover, they should provide direct care on a full-time basis to as many patients as possible, followed by reflection and assessment of their experiences. As a result, we will gain new evidence on what this practice is capable of, regarding the clinical, humanistic, and financial impact. The essential element that is key for this service to become incorporated and comparable to other already established health care services is the policy piece. It will lay the foundations for the service to be reimbursed through the health plan (CHIF). Finally, clinical pharmacists who want to establish a viable clinical practice need to be supported by innovators and visionaries, experts who will steer and push this practice beyond the boundaries. This is our responsibility and commitment toward our patients.
